# Supplementary material for: Association between oral microbiome and seven types of cancers in East Asian population: a two-sample Mendelian randomization analysis
Source: Front Mol Biosci. 2023 Nov 21;10:1327893. doi: 10.3389/fmolb.2023.1327893 (PMC10702768; doi:10.3389/fmolb.2023.1327893)
Supplement: Supplementary file 1 [file DataSheet1.ZIP › Supplementary Materials/MR plots of saliva/saliva═╝/ovarian cancer/pheno.1099_to_ovarian cancer_leave_one_out.pdf]

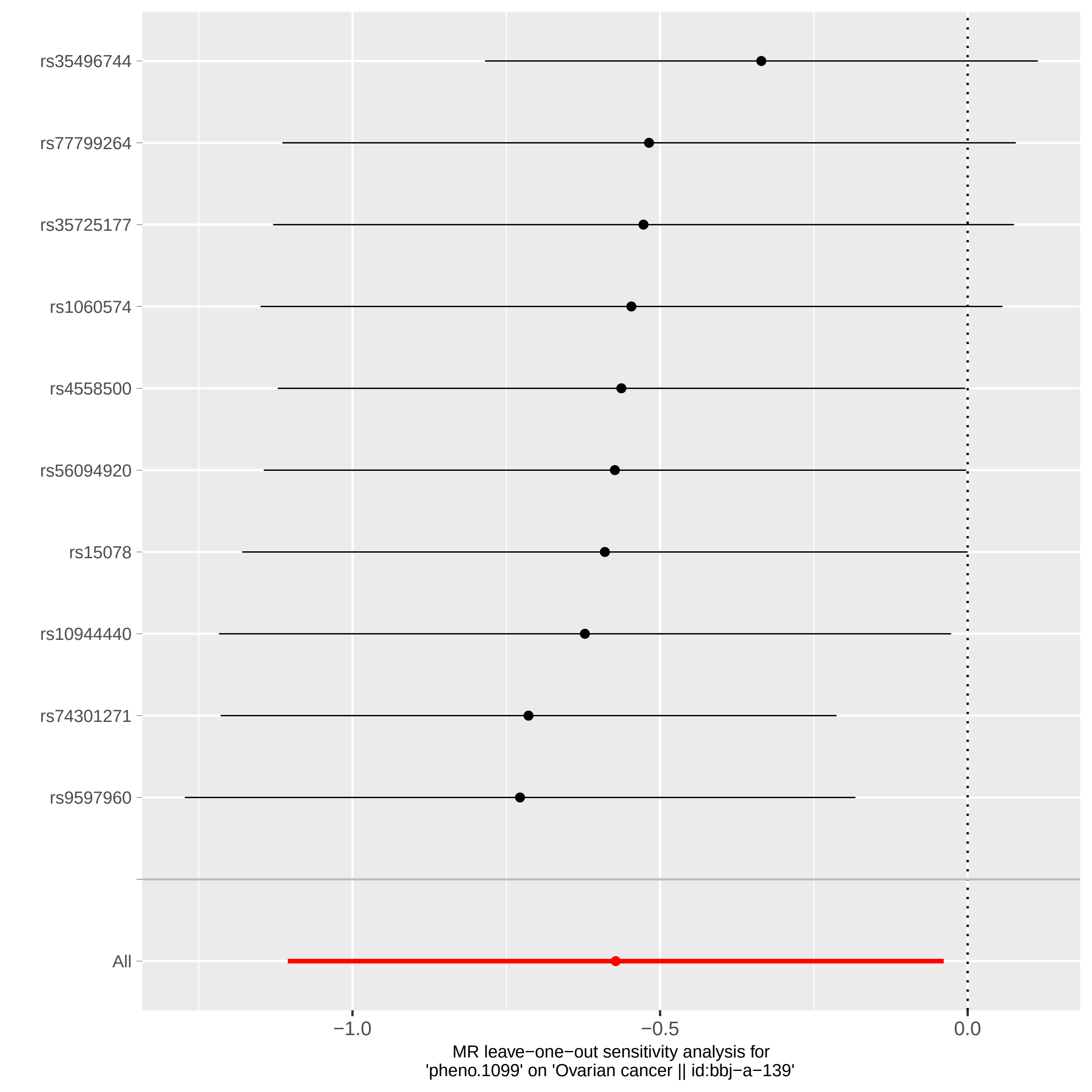
rs35496744
rs77799264
rs35725177
rs1060574
rs4558500
rs56094920
rs15078
rs10944440
rs74301271
rs9597960
All
−1.0
−0.5
0.0
MR leave−one−out sensitivity analysis for
'pheno.1099' on 'Ovarian cancer || id:bbj−a−139'
